# Exploring Uncharted Territory: Toward Metabolite Network Maps

**DOI:** 10.1021/acscentsci.2c01435

**Published:** 2022-12-13

**Authors:** Eva-Maria Dürr, Maja Köhn

**Affiliations:** † Signalling Research Centres BIOSS and CIBSS, University of Freiburg, Freiburg 79104, Germany; ‡ Faculty of Biology, University of Freiburg, Freiburg 79104, Germany

In this issue of *ACS Central Science*, Fiedler and co-workers report a new approach to elucidate the structures of *myo*-inositol polyphosphates (InsPs) and quantify them as part of complex mixtures, which was previously possible only to a very limited extent.^1^ An NMR-based method was developed to characterize compounds arising from the metabolism of InsPs. With structural information on the metabolites in hand, the authors then studied the actions of an enzymatic modulator, the phosphatase MINPP1, on different substances in detail.^1^ Their investigations reveal two distinct metabolic pathways as well as potential mechanisms of regulation and make significant progress toward a fully assigned network of the different inositol phosphate metabolites.

InsPs are small and charged metabolites formed by the phosphorylation of *myo*-inositol and can act as signaling molecules, among other biological roles.^2^ The study of these molecules is challenging due to the presence of multiple isomers, and many current methods rely on radioactive labeling or require separation of the compounds prior to analysis.^3^ Frequently, isolation is based on exploiting the highly charged nature of InsPs, which creates a bias against less charged metabolites such as InsP_2_. Additionally, some InsPs are chiral, and both enantiomers behave identically in conventional purification methods. To overcome these limitations, Nguyen Trung et al. combine ^13^C-labeling of *myo*-inositol with an NMR method that suppresses any signals from protons bound to ^12^C, that is unlabeled material.^4^ This strategy allows for selective monitoring of ^13^C-labeled *myo*-inositol metabolites in a complex sample such as cellular extracts and thereby circumvents the need for isolation of the metabolites of interest. In this study, the 2D HMQC signals of 19 different InsPs and inositol pyrophosphates (PP-InsPs) were assigned and collated in order to identify the metabolites by their NMR signals. This “map” of NMR signals shows clustering dependent on the position of the group and its phosphorylation status, and importantly provides a reference for the assignment of observed signals in samples. As a limitation, unknown products resulting from other pathways may be observed but cannot be assigned yet without a reference spectrum, as was the case for the labeled metabolic extracts of *Schizosaccharomyces pombe*. To resolve the identity of chiral InsPs asymmetrically labeled *myo*-inositol was added to cells, resulting in NMR signals only from the proton attached to the labeled carbon atom(s) (Figure 1a). Using this elegant approach, the identification and stereochemical assignment of poorly characterized chiral InsPs was possible.

**Figure 1:**
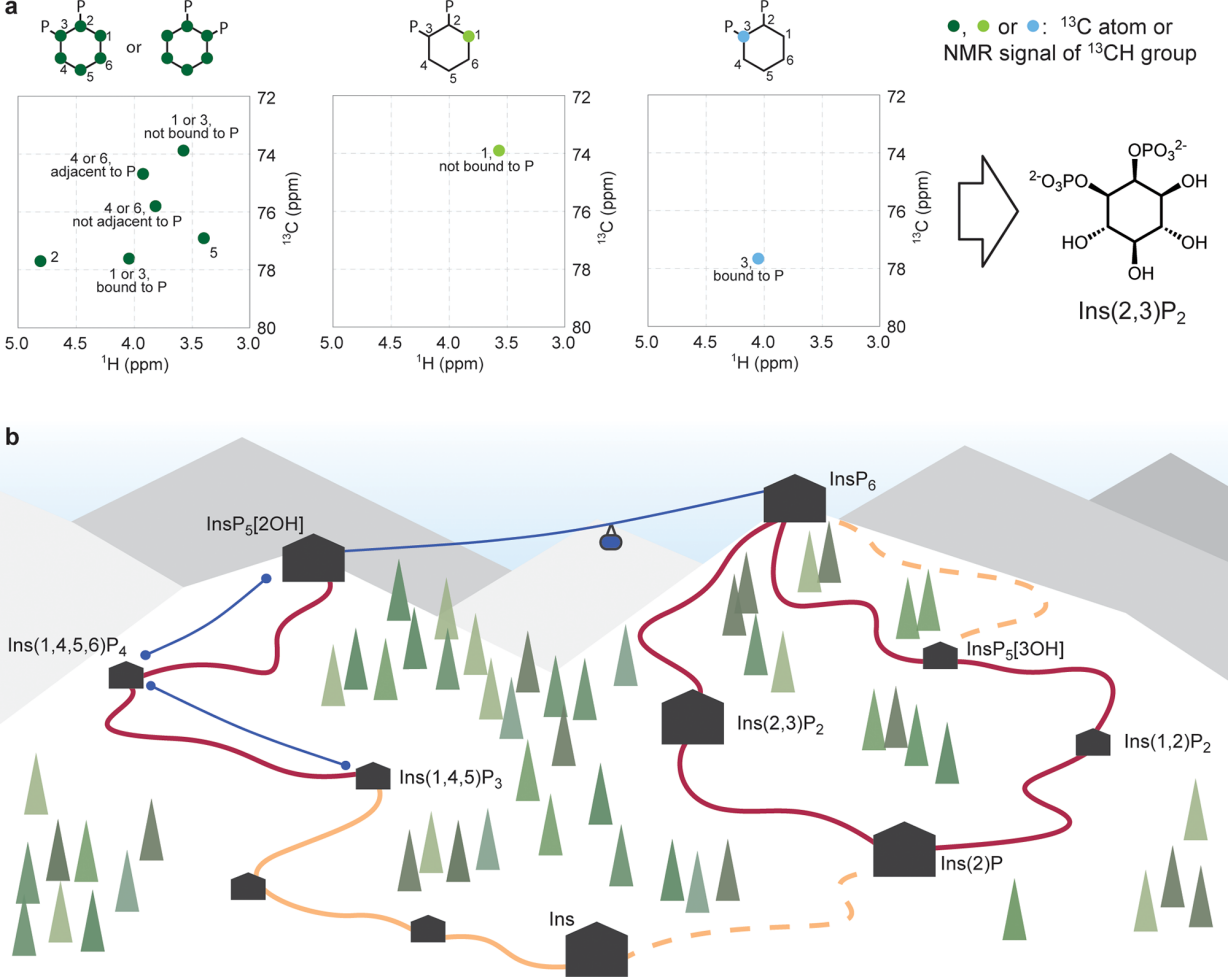
(a) Use of asymmetrically labeled inositol metabolites to assign the structure of Ins(2,3)P_2_. For fully labeled [^13^C_6_]Ins(1/3,2)P_2_, it cannot be determined whether the phosphate group is in position 1 or 3 due to the symmetry. For 1[^13^C]Ins(1/3,2)P_2_, the signal of C1 is observed at the shift corresponding to the unphosphorylated form, whereas for 3[^13^C]Ins(1/3,2)P_2_, the signal of C3 is observed at the shift corresponding to the phosphorylated form. Therefore, the structure can be assigned as Ins(2,3)P_2_. (b) Cartoon representation of the metabolite network of InsPs (black huts; large huts represent metabolites quantified in various cell lines). Phosphorylation reactions catalyzed by known kinases are represented by blue lines (ski lifts). Dephosphorylation reactions catalyzed by MINPP1 are represented as dark red curved lines (slopes), dephosphorylations catalyzed by other phosphatases as orange curved lines (slopes), and dephosphorylations via uncharacterized pathways are represented as dashed curved lines (slopes). Trees and curvature of slopes are for artistic purposes only.

Excitingly, this method is theoretically applicable for studying the metabolism of other types of compounds. A prerequisite for successful assignment is that metabolism of the labeled molecule results in an observable change in chemical shift of a proton attached to a ^13^C atom and that there are no other modifications that result in a highly similar signal. For this to be the case, the metabolic modification needs to be close to the CH group of interest. Additionally, a reference library of known metabolites is necessary to assign chemical shifts. If these requirements can be met, this strategy is a highly promising avenue for fields that are facing similar challenges as the study of InsPs.

The established NMR analysis methodology of ^13^C-labeled metabolites was then applied in this study for the detailed characterization of the only human enzyme known to dephosphorylate InsP_6_, MINPP1.^1^ Previously unassigned metabolites were identified in MINPP1-deficient cells, and InsPs resulting from MINPP1 acting on two different substrates, InsP_5_[2OH] and InsP_6_, were quantified in *in vitro* assays. A potential limitation is the sensitivity of the NMR method, which precludes the assignment of less abundant species. Addressing this issue, experiments requiring higher sensitivity such as kinetic analysis or metabolic flux were carried out by combining the metabolic labeling with capillary electrophoresis coupled to mass spectrometry (CE-MS),^5^ which also distinguishes differently labeled metabolites. By adding differently labeled *myo*-inositol at different time points, the sequence of (de)phosphorylation events in cells could be determined.

The power of the newly developed methodology primarily lies in the detailed structural information it can provide. Importantly, in this work the authors demonstrate how this information can be harnessed to uncover two completely distinct metabolic pathways that both involve MINPP1 (Figure 1b). The generation of Ins(2,3)P_2_ was shown to be dependent on MINPP1 and appeared not to be an intermediate in the biosynthesis of higher InsPs. They also discovered that InsP_6_ acts as a competitive inhibitor, alluding to a potential regulatory mechanism. Regulation of these pathways is of particular interest, since, at least in vitro, MINPP1 is capable of acting on a minimum of 12 substrates, although not all metabolites observed in vitro were observed in cellular extracts. In addition, results from this work point toward the existence of an additional unknown 3-phosphatase that acts on InsP_6_. It is therefore likely that not all of these reactions are catalyzed by MINPP1 in cells. Nonetheless, the multitude of potential reactions catalyzed by MINPP1 and their potential signaling roles indicate that regulation is required. The newly discovered substrate inhibition by InsP_6_, possibly in conjunction with spatial separation, may be one mechanism of directing flux toward certain metabolites. Maintaining the delicate balance between different InsPs is important for cellular function, as an imbalance of InsPs has been linked to mutations of MINPP1 in a subtype of pontocerebellar hypoplasia, a group of early onset neurodegenerative disorders.^6^ Interestingly, the involvement in disease appears to relate to the type of mutations, as different mutations in MINPP1 were suggested to play a role in a subset of malignant follicular thyroid tumors,^7^ while the complete absence of MINPP1 in mice did not lead to a disease phenotype.^8^ The herein established methodologies in combination with CE-MS open up the possibility to identify InsP metabolites with high sensitivity in the presence of different mutations in disease settings, which promises to advance our understanding of the effects of MINPP1 mutations in the disease mechanisms.

Overall, the newly developed methodology enables mapping out the mammalian InsP metabolite network (Figure 1b). A similar analysis in other organisms may require the assignment of additional reference compounds but is now achievable. The detailed characterization of MINPP1 has cleared up questions regarding its substrate scope; at the same time, it raises many questions regarding its regulation and how mutations may affect the dephosphorylation of different substrates, leading to new directions in this challenging research area.

## References

[ref1] Nguyen Trung M.; Kieninger S.; Fandi Z.; Qiu D.; Liu G.; Mehendale N. K.; Saiardi A.; Jessen H. J.; Keller B.; Fiedler D. Stable isotopomers of myo-inositol uncover a complex MINPP1-dependent inositol phosphate network. ACS Cent. Sci. 2022, 10.1021/acscentsci.2c01032. PMC9801504 36589890

[ref2] Irvine R. F.; Schell M. J. Back in the Water: The Return of the Inositol Phosphates. Nat. Rev. Mol. Cell Biol. 2001, 2 (5), 327–338. 10.1038/35073015. 11331907

[ref3] Marolt G.; Kolar M. Analytical Methods for Determination of Phytic Acid and Other Inositol Phosphates: A Review. Molecules 2021, 26 (1), 174 10.3390/molecules26010174. PMC7795710 33396544

[ref4] Harmel R. K.; Puschmann R.; Nguyen Trung M.; Saiardi A.; Schmieder P.; Fiedler D. Harnessing ^13^ C-Labeled *Myo* -Inositol to Interrogate Inositol Phosphate Messengers by NMR. Chem. Sci. 2019, 10 (20), 5267–5274. 10.1039/C9SC00151D. 31191882 PMC6540952

[ref5] Qiu D.; Wilson M. S.; Eisenbeis V. B.; Harmel R. K.; Riemer E.; Haas T. M.; Wittwer C.; Jork N.; Gu C.; Shears S. B.; Schaaf G.; Kammerer B.; Fiedler D.; Saiardi A.; Jessen H. J. Analysis of Inositol Phosphate Metabolism by Capillary Electrophoresis Electrospray Ionization Mass Spectrometry. Nat. Commun. 2020, 11 (1), 6035 10.1038/s41467-020-19928-x. 33247133 PMC7695695

[ref6] Ucuncu E.; Rajamani K.; Wilson M. S. C.; Medina-Cano D.; Altin N.; David P.; Barcia G.; Lefort N.; Banal C.; Vasilache-Dangles M.-T.; Pitelet G.; Lorino E.; Rabasse N.; Bieth E.; Zaki M. S.; Topcu M.; Sonmez F. M.; Musaev D.; Stanley V.; Bole-Feysot C.; Nitschké P.; Munnich A.; Bahi-Buisson N.; Fossoud C.; Giuliano F.; Colleaux L.; Burglen L.; Gleeson J. G.; Boddaert N.; Saiardi A.; Cantagrel V. MINPP1 Prevents Intracellular Accumulation of the Chelator Inositol Hexakisphosphate and Is Mutated in Pontocerebellar Hypoplasia. Nat. Commun. 2020, 11 (1), 6087 10.1038/s41467-020-19919-y. 33257696 PMC7705663

[ref7] Gimm O.; Chi H.; Dahia P. L.; Perren A.; Hinze R.; Komminoth P.; Dralle H.; Reynolds P. R.; Eng C. Somatic Mutation and Germline Variants of MINPP1, a Phosphatase Gene Located in Proximity to PTEN on 10q23.3, in Follicular Thyroid Carcinomas. J. Endocrinol. Metab. 2001, 86 (4), 1801–1805. 10.1210/jcem.86.4.7419. 11297621

[ref8] Chi H.; Yang X.; Kingsley P. D.; O’Keefe R. J.; Puzas J. E.; Rosier R. N.; Shears S. B.; Reynolds P. R. Targeted Deletion of Minpp1 Provides New Insight into the Activity of Multiple Inositol Polyphosphate Phosphatase In Vivo. Mol. Cell. Biol. 2000, 20 (17), 6496–6507. 10.1128/MCB.20.17.6496-6507.2000. 10938126 PMC86124

